# Managing engagement among public, private and civil society actors participating in NewTools: a research partnership on food profiling

**DOI:** 10.1017/S1368980025100621

**Published:** 2025-07-07

**Authors:** Anne Lene Løvhaug, Lisa Garnweidner-Holme, Marianne Hope Abel, Helen Engelstad Kvalem, Kaja Lund-Iversen, Helle Margrete Meltzer, Hanne Fjerdingby Olsen, Laura Terragni, Arnfinn Helleve

**Affiliations:** 1 Institute for Nursing and Health Promotion, Oslo Metropolitan University, Oslo, Norway; 2 Department of Physical Health and Ageing, Norwegian Institute of Public Health, Oslo, Norway; 3 Department of Research Administrative Support, Norwegian Institute of Public Health, Oslo, Norway; 4 Department of Food Safety, Norwegian Institute of Public Health, Oslo, Norway; 5 Faculty of Biosciences, Department of Animal and Aquacultural Sciences, Norwegian University of Life Sciences, Ås, Norway; 6 Centre for Evaluation of Public Health Measures, Norwegian Institute of Public Health, Oslo, Norway

**Keywords:** Food systems, Governance, Stakeholders, Cross-sector partnerships, Framework for engagement

## Abstract

**Objective:**

Partnerships between public, private and civil society actors can potentially address food systems challenges. However, such cross-sector partnerships may require the management of potential tensions and conflicts of interest. This article presents the development and content of a framework for engagement between food systems actors involved in NewTools, a cross-sector research partnership involving twenty-eight partners from research institutions, government, food industry and civil society. The purpose of the framework is to facilitate engagement of partners and maintain research integrity.

**Design:**

This two-phased, iterative study was conducted in 2022. It was guided by recommendations for methodological framework development and was informed by existing frameworks and recommendations as well as two rounds of consultations with partners.

**Setting:**

The Norwegian cross-sector research partnership NewTools that aims to develop two food profiling models: one for dietary quality and one for environmental and social impact.

**Participants:**

Food systems actors involved in the NewTools project.

**Results:**

The NewTools framework consists of four main parts: (1) definition of overarching principles for collaboration (transparency, regular information, adhering to defined roles and responsibilities), (2) descriptions of roles and responsibilities of the partners involved, (3) procedures to ensure involvement and transparency and (4) identification and mediation of potential conflict areas.

**Conclusions:**

This article provides an example of how a cross-sector research partnership developed a framework to facilitate engagement between partners with different interest within a food system. Future studies are needed to assess the potential value of frameworks for cross-sector research partnerships towards healthier and more sustainable food systems.

The disease burden caused by poor diets and negative environmental impacts of food production is substantial^(1)^. There is a need for a shift towards a more equitable food system enabling healthier diets and lower environmental impacts, both to reach the sustainability development goals and improve population health^(2)^. Such a shift requires efforts from all food systems actors, including civil society, the food industry (e.g. primary producers, food manufacturers and retailers) and governmental authorities^(2)^.

Concurrently, processes of economic globalisation have caused a power shift from national governments to international institutions and market actors^(3)^. This has resulted in new forms of collaborative governance including *cross-sector partnerships*, defined here as voluntary, formalised arrangements where two or more societal sectors (public, private and civil society) collaborate towards societal goals^(4,5)^. Because cross-sector partnerships mobilise complementary resources from different actors, they are considered a potential model for addressing complex problems that cannot be solved by single actors or sectors alone^(4–6)^. A range of influential actors, including United Nations fora and research funders, actively encourage cross-sector partnership and collaboration to address food systems challenges^(5,7–10)^. Consequently, there has been a rapid growth of cross-sector partnerships at international as well as national levels^(5)^. These take place within policy, practice and research and address different goals, for example, implementing public policies, delivering services or generating knowledge^(4,6,11)^. The involvement of research institutions into cross-sector partnerships represents unique challenges. Researchers and research institutions should not commit themselves to partnerships that compromise with the basic principles of research such as the integrity of knowledge, collegiality, honesty, objectivity and openness^(12,13)^. In this article, *cross-sector research partnerships* are formalised partnerships involving at least two societal sectors in addition to research institutions, aligning with the specific requirements for research and where the value of scientific evidence is acknowledged as relevant for policy and practice.

Cross-sector partnerships have seemingly become important governance models in public health nutrition policy, practice and research^(4,8)^. However, several public health scholars and organisations including the World Health Organization question the legitimacy of governance models where power is distributed among actors with widely varying interests, mandates and power. In particular, critics address the advisability of partnerships addressing unhealthy diets and non-communicable diseases^(3,7,8,11,14,15)^. The involvement of the food industry, with its commercial interests in unhealthy foods, is seen as a key challenge due to potential conflicts of interest (COI) that may compromise work towards public health^(8,11,14)^. The concept of ‘the commercial determinants of health’, referring to corporate strategies and practices that influence public health, suggests that cross-sector partnerships can be used by the food industry to position themselves as a part of the solution but also to interfere, soften and delay policy development to further their interests^(3,7)^. Influencing science is another commercial determinants of health practice used to promote industry interests^(3,7,16)^. Finally, involvement of the food industry may be challenging due to its considerable instrumental, structural and discursive power, which may enhance its ability to influence processes and public health outcomes^(3,17)^. Thus, cross-sector partnerships addressing healthy diets need to consider how to involve stakeholders with varying interests, mandates and levels of power while also safeguarding outcomes, integrity and credibility^(4,7,8,12,15)^.

A proposed solution to these challenges is to formalise cross-sector partnerships using frameworks that articulate aims and principles that promote engagement while simultaneously preventing COIs^(12,18–21)^. While there is no single, established ‘gold standard’ framework to guide cross-sector partnerships in public health nutrition, several recommendations can be derived from existing academic and policy literature. These include (i) undertake a *risk assessment* process in the planning phases of a partnership to consider risks and benefits for all partners^(12,19,20,22)^; (ii) plan the *composition of a partnership* so that no single actor unduly influences processes and outcomes^(12,18,20,22)^; (iii) set *common goals* ^(8,12,18,19,22)^; (iv) establish *governance structures* that set out roles, mandates, accountability and that identify and manage COIs^(8,12,18,23,24)^ and (v) having in place *systems for transparency and communication* ^(12,20,23,25)^. There is, however, a lack of real-world examples operationalising these^(12)^. Many partnerships have legal contracts that define governance structures and partners’ roles and responsibilities^(19)^, but the formal language of legal contracts is not easily transferable or applicable to day-to-day collaboration in practice^(26)^. A framework describing aims, principles and engagement forms can therefore supplement legal partnership contracts and translate the content into engagement in practice. This article presents the process and the content of a framework for partner engagement in the Norwegian cross-sector research partnership project NewTools.

## The NewTools project – a cross-sector research partnership

NewTools is a four-year cross-sector research partnership aiming to provide concrete tools to support food systems transformation through developing two food profiling models – one for nutritional quality and one for sustainability (i.e. environmental and social impact) – and finally to explore possible applications of these in the Norwegian setting^(27)^. Food profiling models are scientific models developed to assess the nutritional quality and/or social and environmental impact of food products^(28)^ by providing threshold values for nutritional composition and sustainability characteristics. They can be used as the basis for policy actions such as front of pack labelling and public food procurement^(29)^. Obviously, food profiling models can have implications on the commercial interests of food producers, manufacturers and retailers which has for instance been evident in the discourse on front of pack labelling where the implications of food profiling models are debated^(30)^.

The NewTools project was a response to a research call by the Research Council of Norway^(31)^ by the Norwegian Institute of Public Health. According to the funding requirements, non-research sector organisations should be involved as project partners. These should be involved in planning, follow-up and dissemination, as well as being represented in the project’s governance bodies^(31)^. In line with the recommendations for cross-sector partnerships described above, the Norwegian Institute of Public Health independently did the risk assessment, planned the composition and set common goals for the partnership through the development and writing of the project proposal. The risk assessment and planning process did not entail declaration of COI by potential partners, which is often used for ensuring transparency^(12)^. This was because the interests of Norwegian food systems actors, being limited in number, are well known in Norway. Therefore, it was assumed that any COI that might be present were obvious.

The project owner Norwegian Institute of Public Health is a governmental agency and an independent research institution. The project consortium consists of 28 partners representing research institutions, government agencies, food industry (i.e. food producers, manufacturers and retail) and civil society (i.e. interest groups and a consumer organisation) that all have expertise in food and sustainability (Table 1). The project distinguishes between being *collaboration partner* (*n* 13) or *associated partner* (*n* 15). Collaboration partners from research institutions and the consumer organisation are grant beneficiaries, and all collaboration partners including the food industry contribute with their own funding through in-kind work. Associated partners are formally involved in the project without specific obligations and participate at their own costs.

**Table 1.** Partners in the NewTools consortium

Sector	Collaborating partners^*^	Associated partners^†^
Civil society	The Norwegian Consumer Council	Future in Our Hands (environmental organisation)
		EAT Foundation
		Spire (youth environmental and development organisation)
		Matvalget (counseling service for food service operators)
Food industry	Nortura (egg and meat producer)	NorgesGruppen (food retailer)
	TINE (dairy producer and manufacturer)	REMA 1000 (food retailer)
	Felleskjøpet (cooperative for grain and farmer operating assets)	Coop (food retailer)
	Norgesmøllene (grain, flour and baked goods manufacturer)	Orkla (food manufacturer)
	Animalia (meat and poultry research centre)	FoodDrinkNorway (federation for food-, drink and bio industry)
	BAMA (fruit and vegetable importer and manufacturer)	Norwegian Farmers’ and Small Farmers’ Association
		The Norwegian Seafood Federation
Research institutions	Norwegian Institute of Public Health (NIPH)	Institute of Marine Research
	University of Oslo	
	Norwegian University of Life Sciences (NMBU)	
	Norwegian Institute for Sustainability Research (NORSUS)	
	Statistics Norway	
	Oslo Metropolitan University – OsloMet	
Government agencies		Norwegian Directorate of Health
		Norwegian Food Safety Authority
		Norwegian Agriculture Agency

* Consortium members with obligations to engage in project activities and responsibilities to cover costs of their own activities in the project. They have voting rights in the General Assembly.

† Consortium members with opportunities to engage in project activities. They attend the General Assembly without voting rights.

The governance structure of the project is described in the legal consortium agreement,^(32)^ which is signed by all participating parties. This describes the projects’ governance structure, partners’ formal roles and responsibilities, procedural elements and funding details. Online Supplementary File 1 shows the project organisation and describes the roles of the consortium bodies of the project. The consortium agreement states the aims to produce robust and feasible profiling models based on the best available scientific criteria and principles and independent of commercial interests. The agreement also states that project partners have the right to be heard to provide their input and perspectives on the indicators and dimensions, as well as to the feasibility and applicability of the profiling systems. Still, this legal document does not specify *how* the interaction should take place^(32)^.

This article, describing the development and content of a framework for engagement of partners in NewTools, will contribute to the scarce literature on how principles for cross-sector engagement can be operationalised in practice.

## Methods – development of the NewTools framework

The development of the framework for engagement in NewTools was led by researchers and a project coordinator. The development of the framework started as soon as the project period commenced in December 2021 and lasted until June 2022. The process was inspired by the three phases described by McMeekin *et al.* in their recommendations for methodological framework development^(33)^: (i) identifying evidence relevant for the development of the new framework, (ii) adapting the evidence and developing the framework based on the specific context of the NewTools project and (iii) evaluating and refining the framework. This article describes the first two phases, while the evaluation will be reported at a later stage.

### Phase 1: Identification of existing frameworks

According to McMeekin *et al.*, the first phase of framework development entails identification of *existing frameworks or guidance* to form the basis for the new framework^(33)^. In addition, *new data* can be identified to help inform the framework development. This new data can be collected through, for example, literature searches, qualitative research or researcher expertise^(33)^.

NewTools researchers identified two main documents to form the basis of the new framework. First, the NewTools consortium agreement was considered a necessary scaffolding, as it outlined formal governance arrangements that the new framework had to align with. Second, based on researcher’s knowledge, the WHO’s Framework for Engagement with Non-State Actors (FENSA)^(21)^ was chosen to further inform structure and content of the new framework. FENSA was considered relevant as it is an established policy document aiming to strengthen WHO’s engagement with non-state actors while protecting its work from potential risks such as COI. Since the Norwegian Institute of Public Health is a government agency and research institute, it was considered appropriate to base the framework on a policy document. FENSA was also considered useful as it gives examples of how different forms of engagement can take place.

In line with McMeekin *et al.* ^(33)^, a literature review was conducted to identify additional data that could inform the content of the new framework. This was done alongside the development of the new framework and focused on literature providing recommendations for cross-sector engagement related to nutrition and public health. The search strategy and list of included records are provided in online Supplementary File 2. Findings from this search were actively used in the development process.

### Phase 2: Developing the framework

The second phase of framework development involved adapting the existing guidance and data to develop a new framework which responded to the projects’ specific needs. This is described as an iterative process including several rounds of discussions and refinement^(33)^.

### Establishing the main sections of the framework

In initial discussions, the researchers agreed to organise the new framework around specific content from FENSA,^(21)^ which were considered relevant for NewTools. These included:

(i) An explanation of the rationale for engagement between different partner groups including both benefits and risks (in FENSA this is provided in sections 1, 2, 6 and 7). This was considered useful to justify the need for a framework among diverse project participants.

(ii) A description of overarching principles for engagement (inspired by FENSA section 5). It was considered relevant to define a set out cross-cutting principles which would be operationalised in other sections of the framework. The principles were developed by adapting the WHO’s principles and considering recommendations from the literature review (e.g.^(8,12,20,34)^).

(iii) Descriptions of different partners (FENSA sections 8–12). Researchers considered that a section describing the participating organisations and their roles in the project was important for providing transparency and for justifying partner’s involvement. This decision was also informed by findings from the literature review stating the importance of clarifying roles and responsibilities^(12,18,23,35)^.

(iv) Specification of different forms of interaction and procedures for how to carry out these (informed by FENSA sections 14–20 and provisions on operational procedure from pp. 20 onwards). As noted above, FENSA was considered useful due to its provision of concrete explanations of how engagement between different actors can be carried out in a transparent manner.

Researchers decided to include two additional items, based on recommendations from the literature review and researcher’s own experience. First, this involved a description of the goals of the NewTools project and the aims of the framework itself, as recommended in e.g.^(12,18,22)^. Second, they decided to add a section to describe potential COIs and other conflict areas in the project and how these could be managed^(12,20,36)^.

Following this, framework drafts were iteratively revised and refined to fit with the specific context of NewTools. This involved discussions among researchers and involvement of the full consortium as described below.

### Consulting the consortium

Consortium members were involved in the development at two time points. In February 2022, the twenty-eight partners received a survey asking open-ended questions about expectations to the project. One question asked for opinions on how the project could facilitate good dialogue. The recipients received an information letter about the purpose of the survey and were asked to represent their organisations when responding. Providing feedback was voluntary. About half of the partners (*n* 17, three each from civil society, research institutions and government agencies and eight from food industry) submitted feedback. The responses underlined the importance of good and regular communication, equal opportunities to get involved, sufficient time to prepare and respond, using different forms for engagement (such as meetings, workshops and giving written feedback) and mechanisms for transparency such as meetings minutes being available.

In May 2022, all twenty-eight partners and members of the advisory boards received the draft framework and were asked to provide feedback. In formulating feedback, the respondents could choose to respond to questions regarding specific parts of the draft (whether the framework’s purpose, suggested forms of engagement and partner’s roles were well described; if respondents thought the framework could be conflict preventative and if respondents missed anything from the draft). Ten invited actors responded (two advisory board members, two government partners, two civil society/interest group partners and four food industry partners). Of these, three had read the draft and had nothing to add to it, while seven submitted specific feedback or suggestions for amendments. Twenty-three different issues were raised, from minor clarifications to questions around how to handle power structures in the project. Pre-defined criteria for assessing input were not established, and decisions were based on consensus through discussions between researchers. In cases where consensus was not reached, the executive board made the final decisions. Input that aligned with the framework’s aims and the principle of transparency was accepted, while input outside of the framework’s scope or that infringed on researchers’ decision-making authority was rejected. Table 2 provides examples of feedback and decisions on whether these led to changes in the framework or not and justifications for decisions.

**Table 2.** Examples of input to the framework from NewTools consortium members and corresponding executive board decisions

Suggestions from consortium members on amendments to drafts	Decisions and justifications
Adding a relevant law (the Competition Act) to the introduction	Accepted, added to introduction
Extending deadlines for meeting notices and submission of written input	Accepted, additional text added to section on types of engagement
Opening for anonymity in written feedback	Declined, in order to ensure transparency input cannot be given anonymously
Adding more detail around the roles and relationships between collaborating and associated partners, including how to address power imbalances	Taken into account, without leading to amendments. Researchers are responsible for considering and balancing input from partners, including not giving a given perspective too much weight. Transparency procedures will enable assessment of the basis for decisions
Revising one of the overarching principles from: *‘When research decisions cannot be made solely on a scientific basis, researchers describe the rationale underpinning decisions*’, to: *‘When research decisions cannot be made solely on a scientific basis, researchers should **suggest alternative rationales in consultation with all partners and members of the project.’***	Declined, in order to ensure scientific integrity
Adding more detail around conflict management, for example, what happens if a partner leaves the consortium	Accepted, text has been added on conflicts being considered on a case-by-case basis by the executive board. It is hard to envisage how conflicts should be managed, and it is important to have flexibility in this regard
Adding (to a section on conflict areas) that conflicts can arise around interpretation of definitions and the scope of the project	Accepted, added to section on conflicts
Adding a provision on dissemination of preliminary results	Declined, research dissemination is covered by the projects’ publication policy

After consultation, consortium members received a list detailing all input and corresponding responses, including whether input led to draft changes and the reasons for such responses. The NewTools’ executive board adopted the framework in late May 2022. The final stage involved minor revisions in Autumn 2022 to enhance readability for an external audience. This process, overseen by the research coordinator and the article’s first author, focused on language editing and did not involve normative content or consortium input.

## Results – content of the NewTools framework

The NewTools framework for partner engagement (the NewTools framework) supplements the project consortium agreement. Unlike the latter’s legalistic language, it is written in a more user-friendly manner^(37)^. The framework is structured around four areas: (i) aim and overarching principles, (ii) roles and responsibilities, (iii) forms of engagement and (iv) conflict identification and handling^(37)^. The core content of the four areas is described below.

### Aim and overarching principles

The background section of the framework sets out the overall goal of NewTools: to develop two food profiling models. The aim of the framework itself is to serve as a practical guide to facilitate engagement among the consortium partners and at the same time ensure scientific integrity and prevent conflicts. Six overarching principles are defined for the partners to align with:

•All project work and activities shall be carried out in a transparent manner.

•All information about project activities and results shall be easily accessible to consortium partners and external parties.

•All partners shall have an equal opportunity to formulate and present their perspectives.

•All partners in the project will act in line with their defined roles and responsibilities.

•All decisions related to the content of the profiling systems shall be made on scientific evidence. If scientific evidence is limited, the researchers will describe the rationale behind the decisions made.

•All partners shall respect the Competition Act and other relevant laws when carrying out tasks and utilising results.

### Roles and responsibilities

The framework lists all involved partners and describes their roles and responsibilities. These are defined according to the four sectors partners represent: research institutions, government agencies, food industry and civil society. In addition, there are differences according to whether the partner has the status of either collaborating partner or associated partner as defined in the consortium agreement.

The roles and responsibilities are described in three levels. On the first level, descriptions of each group’s *expertise and/or interest* relevant to NewTools and when relevant, their potential *roles* in relation to food profiling systems are provided to acknowledge their relevance in the project. On the second level, all partners’ *opportunities* to provide input and expertise to the project and take part in research that explores the applications of profiling systems are described. The third level, *responsibilities* in the project, mainly lie with the collaborating partners: (1) researchers who are involved in the projects’ work packages and (2) collaborating partners from the food industry and the consumer organisation. Collaborating partners from the food industry are responsible for contributing to the project in-kind but do not receive funding from the Norwegian Research Council. They are expected to provide data on environmental and social sustainability to the project and to contribute with pilots and experiments. The consumer organisation shall provide consumer insights, for example, by conducting surveys or focus group interviews on behalf of the project. Researchers in the project are responsible for gathering and assessing the scientific evidence; collecting and evaluating input and perspectives from other partners and ensuring transparency; and for making decisions on scientific grounds.

### Forms of engagement

The framework describes six different forms of engagement between the project partners, with corresponding procedures to secure transparency: meetings, providing written input, seminars, sharing of data, co-creation (exploration of applications of the food profiling systems) and ongoing dialogue/communication. Actions to secure transparency include: writing minutes of any meeting between researchers and partners/external actors, only allow for written inputs that are open accessible to all (anonymous inputs are not accepted) and provide confirmation of received inputs, how these are considered and responded to. All of these will be stored in a digital platform accessible to the whole project consortium. Other procedures for engagement are that all partners shall receive invitations to give written input with reasonable time to respond and that researchers may involve relevant actors who are not members of the consortium.

### Conflict identification and handling

This section describes that consensus on all matters is neither an expectation nor aim in NewTools. The transparency procedures described above will help identify areas or topics where there are disagreements or potential conflict of interests. The sections on roles and responsibilities and types of engagement are supposed to prevent conflicts and misunderstandings. In addition, acknowledging potential conflict areas may itself be conflict preventative. The framework therefore describes four potential conflict areas in the project: professional disagreement, COIs, conflicts around being heard and conflicts regarding ownership of results.

*(i) Professional disagreement* is expected in NewTools. It is made clear that achieving consensus is not an aim but that all aspects around an issue should be considered before decisions are made. (ii) *Conflict of interests,* particularly those related to partners with financial interests in food profiling systems, are also considered an inevitable aspect of the project. The descriptions of roles and responsibilities, together with transparency measures, are important strategies for managing COIs. COIs can be negative for the consortium’s reputation, independence and credibility. Partners may also experience that their input is not given enough weight or consideration, leading to (iii) *conflicts around being heard*. The procedures for engagement shall ensure that all partners can give input. Importantly, it is stated that not all input will be taken into account, as researchers are responsible for making professional decisions. (iv) *Conflicts on ownership of results* mainly relate to testing of applications of food profiling systems and potential outcomes of these tests. Issues relating to ownership are regulated in the legal consortium agreement.

This section also describes basic mechanisms for conflict mediation. Generally, the project aims to solve conflicts within the relevant work package. If necessary, conflicts can be lifted to the executive board. In the worst case, for instance, if a partner publicly undermines the project or wishes to withdraw due to disagreement, the consortium agreement governs exit procedures. Procedures for conflict mediation were described in such an open manner to maintain flexibility and the possibility to learn and adapt during the project period.

## Discussion

In this article, we have presented the development and content of the NewTools framework for partner engagement. The framework is a supplement to the consortium agreement and contains more detailed descriptions of processes and interactions. Moreover, it provides procedures to ensure transparency on processes and interactions. Theoretical recommendations for cross-sector partnerships already exist^(18,20,22)^ and some have been published after this study was conducted^(13,14)^. To the best of our knowledge, however, this article is novel in that it describes the application of such guidance in practice^(12)^. This may hold relevance for stakeholders interested in nutrition-related cross-sector partnerships, given the steady increase in their number^(5)^. In many contexts, collaboration and partnerships may be a preferred or even mandatory mode of governance^(38)^. Furthermore, despite the global nature of food systems, there are many nutrition-related policy actions that are governed at a national level and where cross-sector partnerships can serve as a potential model for moving policies forward. These include food reformulation, marketing and, to some extent, labelling^(39)^. Due to the contestation around these governance models, it is important to understand how they work under different conditions. In this respect, the NewTools research project can be viewed as an experiment providing such insights.

One of the reasons why cross-sector partnerships are debated is the risk that COI can impair processes and outcomes^(8,13,14,36)^. The commercial determinants of health-related literature has highlighted problematic industry practices such as interference in policy development and research^(3,7,16,40)^, leading to questions of whether the food industry is a legitimate partner in cross-sector collaborations^(41–43)^. Furthermore, food profiling models and related policies like front of pack labelling are contested public health measures that have met stark opposition in recent policy processes^(30,44)^. Given that NewTools is led by a government agency and is closely related to policy, the project may be perceived as policy development more than a research project. A motivation for participation in NewTools might be to gain access to Norwegian and European Union policy processes concerning food profiling models and front of pack labelling^(3,44)^.

Due to the nature of cross-sector partnerships, a framework for engagement can arguably not prevent all the challenges above. Cross-sector partnerships provide arenas for policy dialogue among different stakeholders^(5,6)^. This can be considered as a benefit contributing to shared understanding and improved analysis of a situation before action is taken^(5,6)^, or as a risk of ‘corporate capture’ and COI^(36,40,45)^, depending on the perspective. The framework for engagement in NewTools attempts to bridge these perspectives by enabling partners to provide their expertise to benefit project outcomes, while also safeguarding research integrity through mitigating risks. In line with recommendations for cross-sector engagement^(8,12,18,19)^, the framework clearly defines NewTools as a research project and states that the common goal of the NewTools project is the development of two food profiling systems. The framework also emphasizes the scientific evidence, complementing the project proposal and the consortium agreement. Still, there will likely be disagreements due to divergent positions of partners. The NewTools framework states that the partners will mainly give input to the development of food profiling systems with no promise of shaping results. Such consultancy is considered less problematic than direct involvement in decision-making processes^(14,46,47)^. Nevertheless, the presence of vested interests necessitates COI management, in particular transparency, to enable assessment of processes and outcomes^(12,23,34)^. The framework describes the routines for providing transparency, including how the scientific researchers in the project must respond in writing to justify decisions. Importantly, the NewTools framework is not focused on securing consensus, but rather serve as a mechanism to identify disagreements and differences in opinions.

It has to be acknowledged that the NewTools project takes place in Norway which has traditions for cross-sector engagement, also within nutrition policy. For example, governmental authorities have engaged with the food industry in work towards the Keyhole food labelling scheme; on restrictions on the marketing of unhealthy food and beverages to children^(48)^ and in public–private partnerships to improve the dietary intake of the population^(49)^. Whereas these have been subject to limited academic scrutiny^(50)^, the Norwegian context may be more conducive for cross-sector research partnerships compared to settings where relations between societal sectors are less common or more conflictual. Nevertheless, some of our early experiences from engaging with partners in the framework development process can be noted. For example, it seemed that responding to the framework development was not considered a key priority for all partners. This is perhaps not surprising given that participation is voluntary. Further, the consultations indicated that some partners envisaged taking an active role in research decisions. This required clear communication around the importance of research integrity and preventing COIs. We also found that some responses, such as worries around power imbalances, were challenging to respond to as the project composition was already set. This article will be followed up by studies exploring experiences from the NewTools project, which will contribute to further understanding of barriers and enablers of cross-sector partnerships within the Norwegian setting.

The strengths of this study include the use of recommendations for methodological framework development to guide the process^(33)^. This provided a structured yet flexible process that was well suited for a project facing time restrictions. Also, the involvement of project partners in the development may have contributed to an operational framework that is relevant for participants with diverse capacities, interests and research experience. However, this study also has methodological limitations. First, the framework had to be finalised within a short timeframe. There was therefore not time to complete a systematic literature review ahead of the development, which might have been ideal. However, a purposeful literature review was conducted alongside the development process. We actively used key findings from the literature review to complement the use of WHOs FENSA as our point of departure, for example, by adding sections describing the goals of the project and potential COIs. Second, the framework development process was led by project researchers who made several decisions based on their own expertise, rather than predefined criteria. In this article, we have strived to report the basis for decisions. Third, as described above, not all partners provided feedback on the framework. More involvement to the development process may have strengthened engagement and ownership among partners. Finally, this article excludes considerations taken in the planning stages of the project, for example, important decisions relating to composition of partners and governance bodies. We acknowledge that it may have been beneficial to undertake project planning and framework development in parallel. This might have resulted in inclusion of more participants from civil society to strive for more balanced representation of different sectors^(13,18,20,22)^.

Monitoring and evaluation are necessary to assess whether a framework for engagement will achieve its aims^(20,25,46)^. There are several possibilities for doing this. For example, one of the overall aims of the framework is to facilitate engagement. A survey complemented by qualitative interviews can explore how partners experience collaboration. Furthermore, it will be important to assess whether researchers document their engagement activities transparently. This can be done by appraising if, for example, meeting minutes are stored on the shared digital platform. Such transparency implementation will be followed up upon through regular meetings and will be the responsibility of specific researchers in NewTools. However, assessing the extent to which the framework has met its aim to ensure scientific integrity should be judged by independent actors and the research community^(36)^.

### Conclusion

The NewTools framework for engagement is a novel example of how a cross-sector research partnership have operationalised principles for collaboration. The framework consists of four main parts: (1) defined overarching principles for collaboration (transparency, regular information, adhering to defined roles and responsibilities), (2) descriptions of roles and responsibilities of the partners involved, (3) procedures to ensure involvement and transparency and (4) identification and mediation of potential conflict areas. Whether in policy, practice or research, cross-sector partnerships need to manage stakeholder engagement to secure the trustworthiness of processes and outcomes. By incorporating overarching principles, clear role descriptions, transparent procedures and conflict mediation approaches, the NewTools framework provides an actionable foundation for the involvement of partners while also securing research integrity. Articles like this, containing descriptions of the development of frameworks for engagement in cross-sector partnerships may benefit decision makers and guide future work where collaboration is the preferred form of governance. Further research is needed to assess whether frameworks for engagement function as intended.

## Supporting information

Løvhaug et al. supplementary material 1 Løvhaug et al. supplementary material

Løvhaug et al. supplementary material 2 Løvhaug et al. supplementary material

## Supplementary material

For supplementary material accompanying this paper visit https://doi.org/10.1017/S1368980025100621
