# Supplementary material for: Managing engagement among public, private and civil society actors participating in NewTools: a research partnership on food profiling
Source: Public Health Nutr. 2025 Jul 7;28(1):e116. doi: 10.1017/S1368980025100621 (PMC12426873; doi:10.1017/S1368980025100621)
Supplement: Løvhaug et al. supplementary material 1 — Løvhaug et al. supplementary material [file S1368980025100621sup001.docx]

**Supplementary file 1. Consortium organization in the NewTools project**


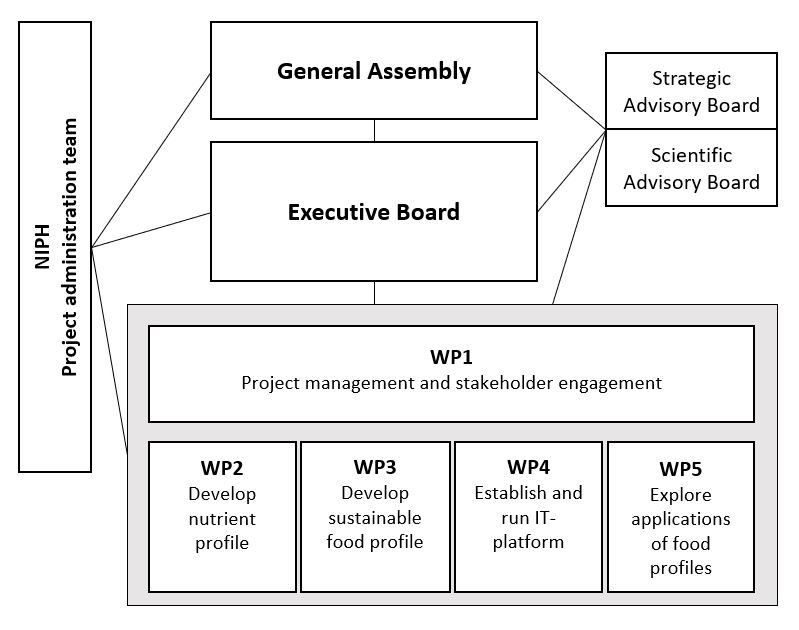


Figure 1. Project organization in the NewTools consortium. WP = Work packages. NIPH: Norwegian Institute of Public Health

Table 1. Consortium bodies of the NewTools project

| **Consortium bodies** | **Tasks and composition** |
| --- | --- |
| The General Assembly | The consortium body where all project partners can meet is held annually. It makes decisions on administrative issues (e.g. consortium composition, identification of breach of consortium agreement and remedies and termination of defaulting parties). It does not make professional or science-related decisions. There is one representative from each partner. Collaborating partners have voting rights, while associated partners do not have voting rights. |
| Executive Board | It is responsible for the monitoring and managing of the progress of the project. Members: Project manager and work package leaders. |
| Scientific Advisory Board (external members) | It will advise on the direction of the project, ensure that project results are relevant and inform about potential risks and opportunities.  Members: Five external independent experts: Two from academia, two from foundations for product labelling, and one from a nutrition and diet association. |
| Strategic Advisory Board | The Strategic Advisory Board shall contribute to the project progressing according to plans, ensure that different views are included in the process and that the project impact is maximized in Norway and internationally. Members: four collaboration partner representatives and one external representative (from a consulting company within sustainable agriculture) in addition to the NIPH Director-General, the project manager, and work package leaders. |
| NIPH Project Administration Team | Assists and facilitates the work of the consortium bodies as well as the day-to-day management of the project. |
