# Supplementary material for: Managing engagement among public, private and civil society actors participating in NewTools: a research partnership on food profiling
Source: Public Health Nutr. 2025 Jul 7;28(1):e116. doi: 10.1017/S1368980025100621 (PMC12426873; doi:10.1017/S1368980025100621)
Supplement: Løvhaug et al. supplementary material 2 — Løvhaug et al. supplementary material [file S1368980025100621sup002.docx]

**Supplementary file 2. Identification of additional guidance for the framework development**

To identify relevant, additional recommendations on stakeholder engagement we conducted a purposeful literature review in January 2022. In this supplementary file we provide the search strategy and the resulting list of included literature.

# Search strategy

Assisted by a university librarian, we developed a search strategy for a purposeful literature review of scientific and grey literature. The searches were performed by the first author.

The search for **academic literature** was conducted in January 2022 in the Medline, Embase (OVID interface) and Scopus databases, with search terms tailored for each. Search terms within four thematic areas were combined with OR and the thematic areas were combined with AND (Table 1). Search strings for each database is available upon request. We limited the search to English or Scandinavian language articles published after 2015 to capture recent literature and considering that WHO FENSA (1) was published in 2015.

Table 1. Examples of search terms

| **Thematic areas**  Combined with AND | **Search terms**  Subject headings and text searches for title [ti] and authors’ key word headings [kw and kf], combined with OR |
| --- | --- |
| Field of research | International agencies, nutrition science, public health research |
| Sectors | Civil society, food industry, non-governmental, private sector, public sector |
| Partnership | Cooperation, involvement, multistakeholder, public-private partnership |
| Area of partnership | Diet, food, food system, population health, public health, sustainable food |
| Recommendations | Advice, conflict of interest, consensus, framework, funding, governance, guideline, involvement, participation, policy, principle, recommendation, research ethics, vested interest |

The search strategy for **grey literature** (i.e., policy documents) was informed by the approach of Cullerton et al (2). In January 2022 the first author performed a basic search in Google, using a simplified set of key search terms (nutrition + recommendation + framework + "conflict of interest" + filetype:pdf). A similar search was conducted in Google Scholar.

## Inclusion criteria

Inclusion criteria were set up in advance.

- For **publication type** we included academic journal articles of any kind and grey literature defined as policy documents published by governments, international organizations, professional health/public health organizations or scientific expert bodies. We excluded books, conference procedures and presentations.
- For **content**, we included records that provided “frameworks”, broadly conceptualized as recommendations, guidelines, advice etc. that related to engagement between different societal stakeholders in the areas of public health, food, nutrition, or food related sustainability and in the context of policy/governance and/or research. We excluded resources containing general discussions or critique of stakeholder engagement that did not suggest any own recommendations.

## Screening

The screening process was performed by the first and second author.

**Database searches:** Academic journal articles were imported to the Endnote X9 reference manager system where duplicates were removed. An initial screening based on title was performed by the first author who excluded records that were not thematically relevant and exported the remaining records to the Rayyan software service (3) for further screening. The first and second author then screened records based on abstracts. They excluded records that did not present frameworks or recommendations on stakeholder involvement. Finally, they assessed the full texts of all remaining records and excluded those that did not meet all inclusion criteria.

**Grey literature searches:** in line with Cullerton’s (2) approach, the first 50 hits from the basic Google search were retrieved for screening. For the Google Scholar screening, the first 50 hits were scanned immediately by the first author and only records that had not been identified in the basic Google search were retrieved. The records from both searches were saved for further screening in a digital folder and screened by both authors against inclusion criteria.

**Additional searches:** To identify additional relevant papers the first author inspected the reference lists of all included journal articles and conducted citation searches of three key articles in Web of Science, Scopus and Google Scholar. Results were screened in full text by both authors. Any discrepancies were solved by discussion.

## Data extraction

The following data was extracted from the included records into an Excel sheet: reference; publication year; document type (journal article + article type or grey literature); aim of document as stated by document’s authors; and information about funding and COI. The data extraction sheet is available upon request.

## Analysis

Included records were uploaded to the NVivo software (QSR International Pty Ltd. (2020) NVivo (released in March 2020), <https://www.qsrinternational.com/nvivo-qualitative-data-analysis-software/home>) to facilitate categorization of recommendations for engagement between stakeholders.

# Results


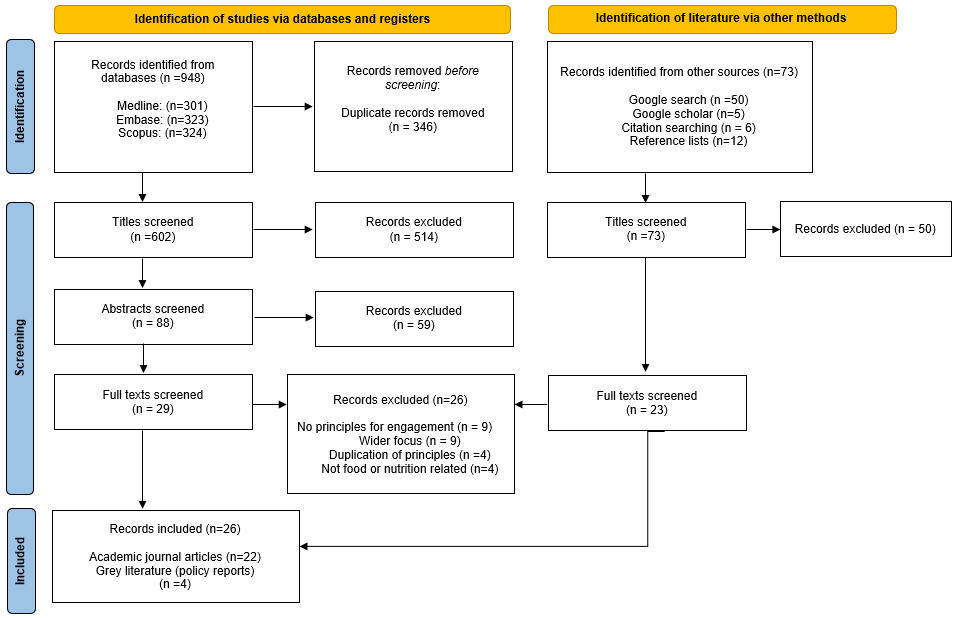
After screening (PRISMA flow chart, Figure 1), we included 22 journal articles and four policy reports containing recommendations, principles and advice for engagement between stakeholders within public health, food and nutrition published after 2015.

Figure 1. PRISMA flowchart. From: Page MJ, McKenzie JE, Bossuyt PM, Boutron I, Hoffmann TC, Mulrow CD, et al. The PRISMA 2020 statement: an updated guideline for reporting systematic reviews. BMJ 2021;372:n71. doi: 10.1136/bmj.n71.

## List of included records

1. Alexander et al. Achieving a transparent, actionable framework for public-private partnerships for food and nutrition research. Am J Clin Nutr. 2015;101(6):1359-63.
2. Bertolo RF, Hentges E, Makarchuk MJ, Wiggins AKA, Steele H, Levin J, et al. Key attributes of global partnerships in food and nutrition to align research agendas and improve public health. Appl Physiol Nutr Metab. 2018;43(7):755-8.
3. Collins T, Mikkelsen B, Axelrod S. Interact, engage or partner? Working with the private sector for the prevention and control of noncommunicable diseases. Cardiovasc. 2019;9(2):158-64.
4. Cullerton K, Adams J, Forouhi N, Francis O, White M. What principles should guide interactions between population health researchers and the food industry? Systematic scoping review of peer-reviewed and grey literature. Obes Rev. 2019;20(8):1073-84.
5. Cullerton K, Adams J, Francis O, Forouhi N, White M. Building consensus on interactions between population health researchers and the food industry: Two-stage, online, international Delphi study and stakeholder survey. PLoS ONE. 2019;14(8):e0221250.
6. Drewnowski A, Caballero B, Das JK, French J, Prentice AM, Fries LR, et al. Novel public-private partnerships to address the double burden of malnutrition. Nutrition Reviews. 2018;76(11):805-21.
7. Fanzo J, Shawar YR, Shyam T, Das S, Shiffman J. Challenges to Establish Effective Public-Private Partnerships to Address Malnutrition in All Its Forms. International journal of health policy and management. 2021;16.
8. Huang TTK, Ferris E, Crossley R, Guillermin M, Costa S, Cawley J. A protocol for developing an evaluation framework for an academic and private-sector partnership to assess the impact of major food and beverage companies' investments in community health in the United States. BMC Obes. 2015;2(1) (no pagination).
9. Jones A, Neal B, Reeve B, Ni Mhurchu C, Thow AM. Front-of-pack nutrition labelling to promote healthier diets: Current practice and opportunities to strengthen regulation worldwide. BMJ glob. 2019;4(6).
10. Kraak VI. Critique of a scoping review of principles to guide interactions between population health researchers and the food industry. Obes Rev. 2019;20(9):1335-7.
11. Kraak VI. Advice for Food Systems Governance Actors to Decide Whether and How to Engage With the Agri-Food and Beverage Industry to Address Malnutrition Within the Context of Healthy and Sustainable Food Systems Comment on "Challenges to Establish Effective Public-Private Partnerships to Address Malnutrition in All Its Forms". International journal of health policy and management. 2021;06.
12. Mialon M, Vandevijvere S, Carriedo-Lutzenkirchen A, Bero L, Gomes F, Petticrew M, et al. Mechanisms for addressing and managing the influence of corporations on public health policy, research and practice: a scoping review. BMJ Open. 2020;10(7):e034082.
13. Mozaffarian D. Conflict of Interest and the Role of the Food Industry in Nutrition Research. Jama. 2017;317(17):1755-6.
14. Poli A, Marangoni F, Agostoni CV, Brancati F, Capurso L, Colombo ML, et al. Research interactions between academia and food companies: how to improve transparency and credibility of an inevitable liaison. Eur J Nutr. 2018;57(3):1269-73.
15. Ralston R, Hill SE, Gomes FS, Collin J. Towards preventing and managing conflict of interest in nutrition policy? An analysis of submissions to a consultation on a draft who tool. International Journal of Health Policy and Management. 2021;10(5):255-65.
16. Simon C, Kocot SL, Dietz WH. Partnership for a Healthier America: Creating Change Through Private Sector Partnerships. Curr. 2017;6(2):108-15.
17. Swinburn B, Kraak V, Rutter H, Vandevijvere S, Lobstein T, Sacks G, et al. Strengthening of accountability systems to create healthy food environments and reduce global obesity. The Lancet. 2015;385(9986):2534-45.
18. Food and Agriculture Organization of the United Nations (FAO). Briefing note. Management of potential conflicts of interest in nutrition policy. FAO; 2019.
19. Lie AL, Granheim SI. Multistakeholder partnerships in global nutrition governance: protecting public interest? Tidsskrift for Den norske legeforening. 2017.
20. World Health Organization (WHO). Safeguarding Against Possible Conflicts of Interest in Nutrition Programmes. Draft approach on the prevention and management of conflicts of interest in the policy development and implementation of nutrition programmes at country level: Proposed Decision-Making Process and Tool. Geneva: WHO; 2017.
21. Kraak, V.I., and Story, M. 2015. Guiding principles and a decision-making framework

for stakeholders pursuing healthy food environments. Health Affairs (Millwood), 34(11): 1972–1978. doi:10.1377/hlthaff.2015.0635

1. World Health Organization. Guiding principles and framework manual for front-of-pack labelling for promoting healthy diet. Geneva, Switzerland, 2019.
2. World Cancer Research Fund International. Building momentum: lessons on implementing a robust front-of-pack food label. London, UK, 2019.
3. Reeve B, Gostin LO. "Big" food, tobacco, and alcohol: reducing industry influence on noncommunicable disease prevention laws and policies. Comment on ”addressing ncds: challenges from industry market promotion and interferences". Int J Health Policy Manag. 2019;8:450–4.
4. Buse K, Tanaka S, Hawkes S. Healthy people and healthy profits? Elaborating a conceptual framework for governing the commercial determinants of non-communicable diseases and identifying options for reducing risk exposure. Global Health. 2017;13(1):34. doi:10.1186/

s12992-017-0255-3

1. Pérez-Escamilla R. Innovative Healthy Lifestyles School-Based Public–Private Partnerships Designed to Curb the Childhood Obesity Epidemic Globally: Lessons Learned From the Mondelēz International Foundation. Food and Nutrition Bulletin. 2018;39(1_suppl):S3-S21.

# References

1. Resolution WHA69.10. Framework of engagement with non-State actors. Sixty-ninth World Health Assembly, Geneva, 23–28 May 2016. Geneva: WHO 2016.

2. Cullerton K, Adams J, Forouhi N, Francis O, White M. What principles should guide interactions between population health researchers and the food industry? Systematic scoping review of peer-reviewed and grey literature. Obesity Reviews. 2019;20(8):1073-84.

3. Ouzzani M, Hammady H, Fedorowicz Z, Elmagarmid A. Rayyan—a web and mobile app for systematic reviews. Systematic reviews. 2016;5(1):210.
